# Nrf2 Expressions Correlate with WHO Grades in Gliomas and Meningiomas

**DOI:** 10.3390/ijms17050722

**Published:** 2016-05-13

**Authors:** Wen-Chiuan Tsai, Dueng-Yuan Hueng, Chii-Ruey Lin, Thomas C. K. Yang, Hong-Wei Gao

**Affiliations:** 1 Department of Pathology, Tri-Service General Hospital, National Defense Medical Center, No. 325, Sec. 2, Cheng-Kung Road, Neihu 114, Taipei 11490, Taiwan; drtsaiwen@gmail.com; 2 Graduate Institute of Engineering Technology, Collage of Engineering, National Taipei University of Technology, Taipei 10655, Taiwan; ckyang@mail.ntut.edu.tw; 3 Department of Neurological Surgery, Tri-Service General Hospital, National Defense Medical Center, Taipei 11490, Taiwan; hondy2195@yahoo.com.tw; 4 Institute of Mechatronic Engineering and Biotechnology, National Taipei University of Technology, Taipei 10655, Taiwan; crlin@ntut.edu.tw; 5 Department of Chemical Engineering and Biotechnology, National Taipei University of Technology, Taipei 10655, Taiwan

**Keywords:** Nrf2, glioma, meningioma, World Health Organization (WHO) grade

## Abstract

Background: Nuclear factor erythroid 2-related factor 2 (NFE2L2, also known as *Nrf2*) is associated with cellular progression and chemotherapeutic resistance in some human cancers. We tested the relationship between Nrf2 expression and survival of patients with primary brain tumors (PBTs). Methods: In order to realize Nrf2 protein expression in gliomas, Western blot analysis was performed in normal brain tissue and U87MG, LN229, GBM8401 and U118MG glioma cell lines protein lysates. Then, U87MG, LN229, and GBM8401 mRNA were applied to performed quantitative RT-PCR for detect *Nrf2* gene expression in glioma cell lines. At last, immunohistochemical analysis was used to determine the expression of Nrf2 in samples from 178 PBTs and 10 non-neoplastic brain tissues. Results: In these included *in vitro* studies, both Nrf2 protein and mRNA expression in all human glioma cell lines were higher than normal brain tissue. Similarly, on the viewpoint of immunohistochemistry, Nrf2 expression in gliomas were positively correlated with World Health Organization (WHO) grades. Additionally, compared with the expression of Nrf2 in non-neoplastic brain tissue, expression in meningiomas was of a stronger intensity and was present in a higher percentage of cells. Furthermore, scores were significantly higher in WHO grade II than in WHO grade I meningiomas. Finally, overall survival tended to be shorter in patients whose PBTs had higher expression of Nrf2, although the correlation was not statistically significant. Conclusions: Nrf2 overexpression positively correlated with WHO grade in gliomas and meningiomas. On the other hand, Nrf2 immunohistochemical stain could help pathologists to differentiate atypical meningiomas from benign tumors. Therefore, Nrf2 expression may be a useful biomarker to predict WHO grade and cellular behavior of PBTs.

## 1. Introduction

From 2006 to 2010, the incidence of tumors of the primary central nervous system was 27.4 per 100,000 people, as reported by The Central Brain Tumor Registry of the United States [1]. Gliomas and meningiomas, the leading two types of tumors that occur in the intracranial region, comprise 85% of cases of primary brain tumors (PBTs) [2,3]. A recent study suggested that various molecular epidemiologic factors including mutative DNA damage, cell-cycle disarrangement, metabolic disorders, and long-term inflammatory conditions can contribute to the development of PBTs [4]. The interaction between environmental exposure and genetic susceptibility is also viewed as an important factor that can induce cellular proliferation in PBTs [2]. Accordingly, PBT is considered to be a multifactorial disease [4]. Tumor migration and metastasis are two factors that influence survival for patients with glioma [5]. Additionally, the prognosis of meningioma depends on tumor recurrence and aggressiveness [6,7,8]. The World Health Organization (WHO) grading system is the most important tool to evaluate these prognostic factors. Unlike other solid organs in human bodies, the small amount of fragmented specimens of PBTs may cause artificial disorientation because of the restricted surgical space [9]. Vankalakunti *et al.* demonstrated that the discrepancy rate between histological classification and aggressive behavior is approximately 7% to 20% for meningioma [10]. Therefore, the development of new biomarkers is crucial to minimize the difficulty of diagnosis.

Nuclear factor erythroid 2-related factor 2 (NFE2L2, also known as *Nrf2*), a nuclear transcription factor, is the main regulator of intracellular antioxidants and phase II detoxification enzymes [11]. Binding of Nrf2 to the antioxidant-response element (ARE) within the promoter regions of cytoprotective genes shields cells from oxidative injury [12]. The ARE regulates several phase II detoxification and antioxidant genes such as the Kelch-like ECH-associated protein 1 gene (*Keap1*). Keap1 is a member of the Cul3-dependent E3 ubiquitin ligase complex, which induces the degradation of Nrf2 to suppress the expression of genes that are downstream targets of Nrf2 [13]. Therefore, Nrf2 plays an important role in protecting against cardiovascular disease, inflammatory lung disorders and fibrosis, diabetic nephropathy, and even malignant disease [14,15,16,17,18,19,20,21]. However, the results of a recent study indicate that strong expression of Nrf2 is related to chemoresistance in some human cancers [13,22,23,24]. In previous *in vitro* studies, Nrf2 was shown to be associated with the migration and invasion of glioma cells, as well as with renewal of glioma stem cells [11,25]. However, we still lack evidence of Nrf2 expression in human glioma *in vivo*.

In this study, we used immunohistochemical stain and Western blot analysis to determine if Nrf2 expression is related to tumor progression and metastasis in PBTs. Our results successfully demonstrate that higher scores of Nrf2 expression in PBTs are significantly correlated with more advanced WHO grades. To the best of our knowledge, this is the first study to investigate the association between Nrf2 expression and both WHO grade in glioma and meningioma and overall survival (OS) of patients with these tumors.

## 2. Results

### 2.1. Nrf2 Protein Overexpression in Human Glioma Cell Lines

**Figure 1:**
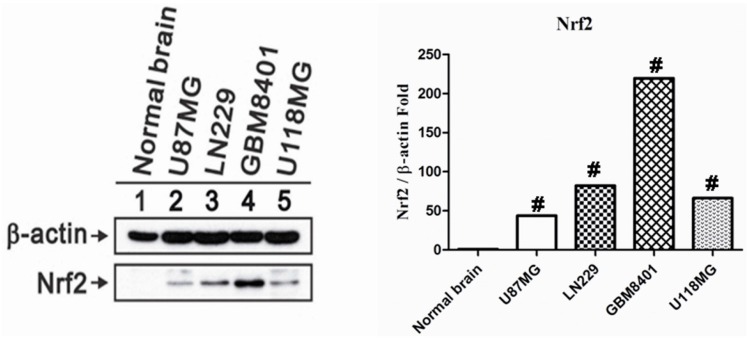
Nrf2 protein Expression in U87MG, LN229, GBM8401 and U118MG human glioma cell lines and normal brain tissue protein lysates. β-actin served as a loading control. # means *p* < 0.01.

Compared with normal brain tissue cell lysate, the Western blot analysis showed higher Nrf2 protein production in U87MG, LN229, GBM8401 and U118MG human glioma cell lines. In the *in vitro* study, we demonstrated the phenomenon of Nrf2 overexpression in all human glioma cell lines (Figure 1).

### 2.2. Higher Nrf2 mRNA Expression in Human Glioma Cell Lines

**Figure 2:**
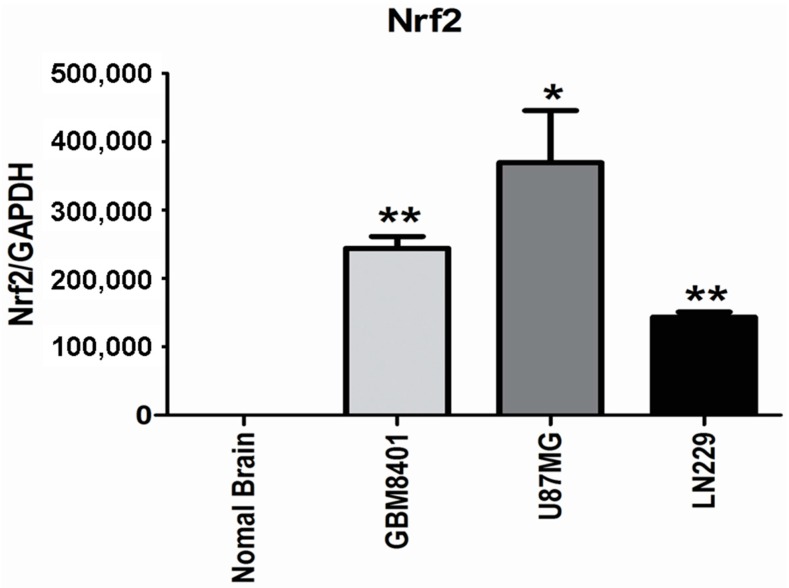
qRT-PCR was performed to examine Nrf2 mRNA expression in U87MG, LN229, GBM8401 and U118MG human glioma cell lines and normal brain tissue. The relative expressions were normalized with normal brain tissue. Bars, means ± SEM; * means *p* < 0.001, ** means *p* < 0.01. Data are representative of three independent experiments.

In order to determine the differences of Nrf2 mRNA expression in human glioma cell lines and normal brain tissues, the low grade glioma cell line, LN229, and WHO grade IV glioma cell lines, U87MG and GBM8401, were applied to perform quantitative RT-PCR. All human glioma cell lines revealed higher expression of Nrf2 mRNA expression than normal brain tissues. In addition, the degree of Nrf2 mRNA expression in U87MG and GBM8401 was higher than LN229 (Figure 2).

### 2.3. Clinicopathological Characteristics in Tissue Microarrays

This study included seven cases of WHO grade I, 50 WHO grade II, 22 WHO grade III, and 13 grade IV glioma, as well as 57 cases of WHO grade I and 12 WHO grade II meningioma. We also analyzed other neuroepithelial tumors including eight cases of central neurocytoma, four of chordoma, and five of medulloblastoma. The case number and tumor differentiation stage of all samples are listed in Table 1.

### 2.4. Nrf2 Expression Correlates with High-Grade Gliomas

**Figure 3:**
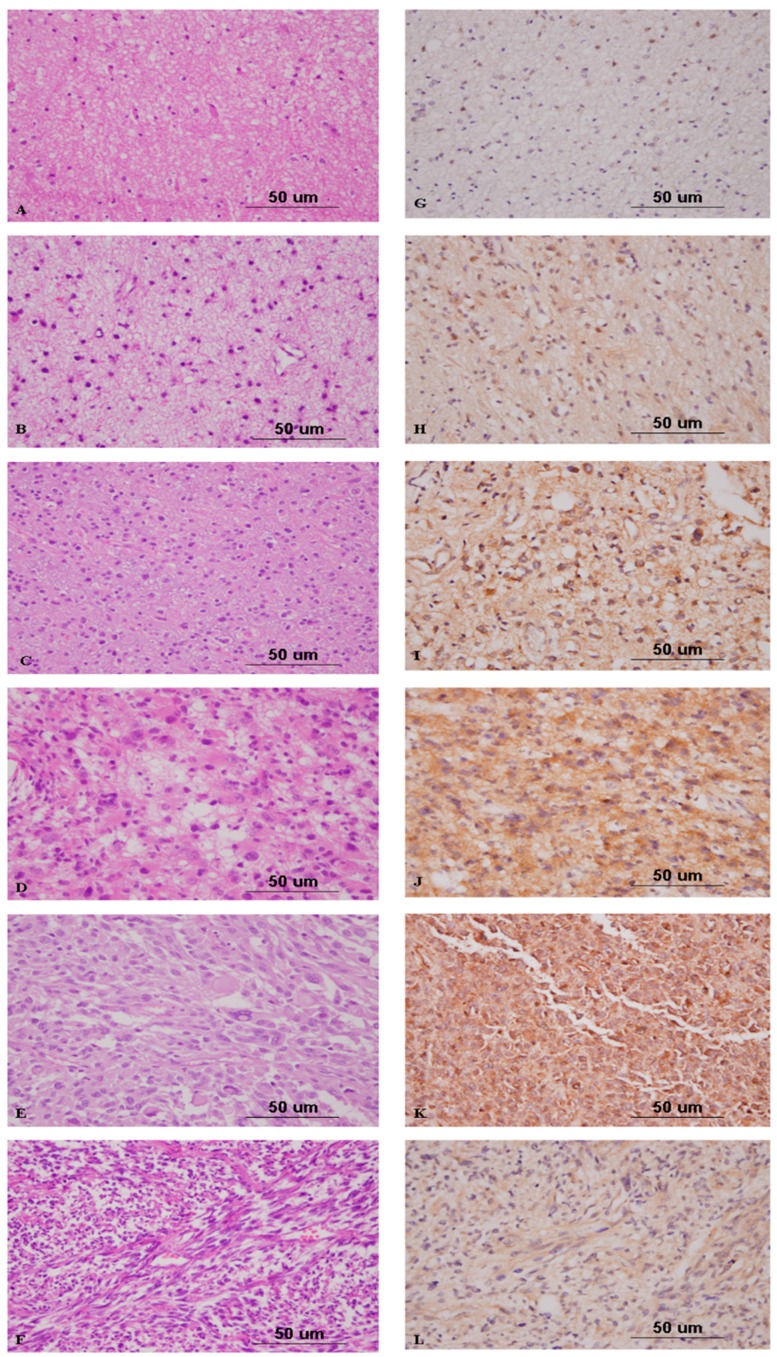
Hematoxylin and eosin staining of non-neoplastic brain tissue (**A**); pilocytic astrocytoma (**B**); diffuse astrocytoma (**C**); anaplastic astrocytoma (**D**); glioblastoma multiforme (**E**); and gliosarcoma (**F**); and immunohistochemical analysis of Nrf2 in non-neoplastic brain tissue (**G**); pilocytic astrocytoma (**H**); diffuse astrocytoma (**I**); anaplastic astrocytoma (**J**); glioblastoma multiforme (**K**); and gliosarcoma (**L**) (original magnification ×400).

**Figure 4:**
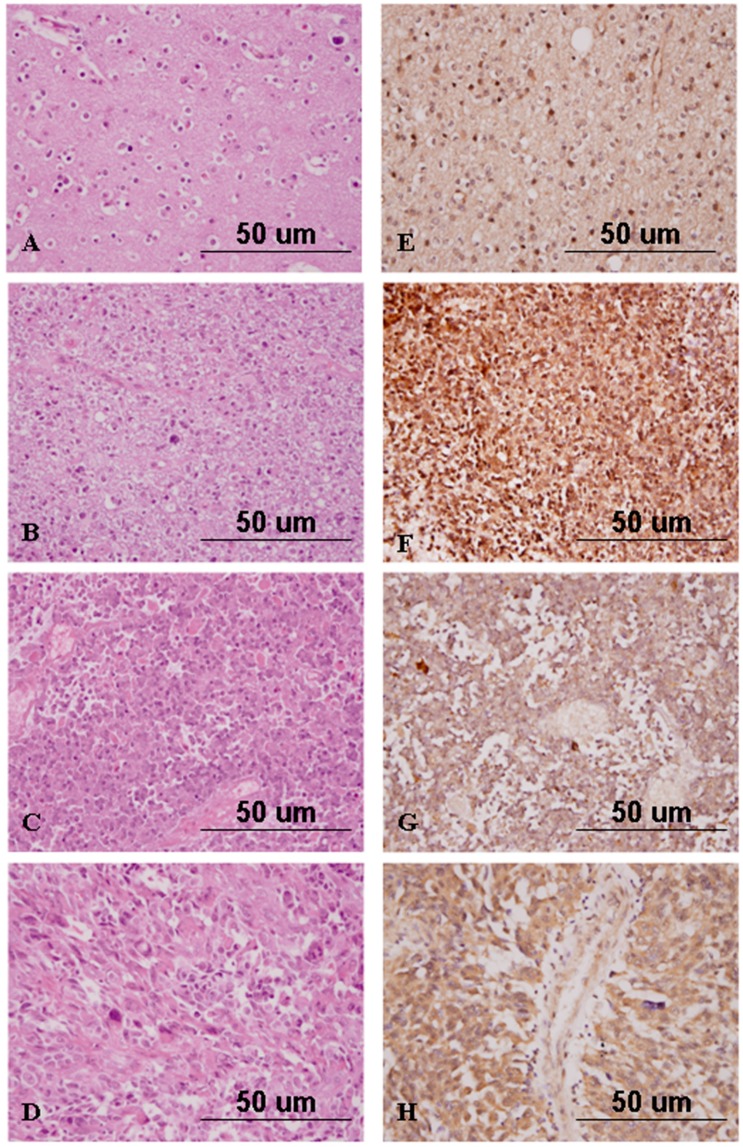
Hematoxylin and eosin staining of oligodendroglioma (**A**); anaplastic oligodendroglioma (**B**); ependymoma (**C**); and anaplastic ependymoma (**D**); and immunohistochemical analysis of Nrf2 in oligodendroglioma (**E**); anaplastic oligodendroglioma (**F**); ependymoma (**G**); and anaplastic ependymoma (**H**) (original magnification ×400).

**Figure 5:**
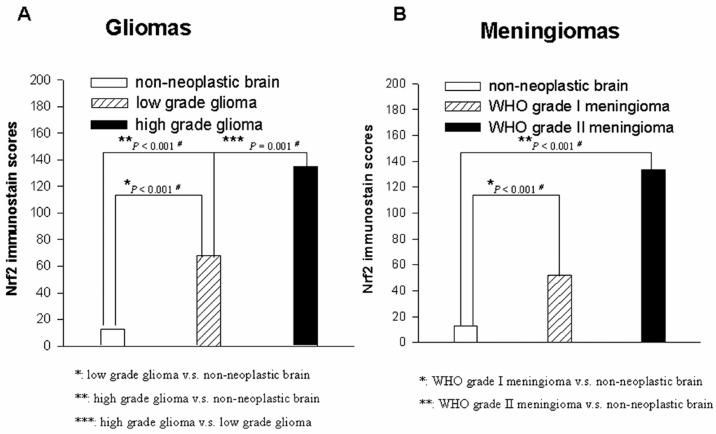
The comparability of Nrf2 imunostain scores in non-neoplastic brain tissue, low grade glioma and high grade glioma (**A**); and the comparability of Nrf2 imunostain scores in in non-neoplastic brain tissue, WHO grade I meningioma and WHO grade II meningioma (**B**).

The expression of Nrf2 in non-neoplastic brain tissues was weaker in intensity and was present in a smaller percentage of cells than was expression in the samples from the PBTs. Among the 72 cases of astrocytic tumors of various WHO grades, the average expression scores of Nrf2 were 19.29 in pilocytic astrocytomas, 75.98 in diffuse astrocytomas, 114.00 in anaplastic astrocytomas, 160.56 in glioblastomas multiforme, and 42.50 in gliosarcomas (Table 2, Figure 3). Statistical analysis revealed a positive correlation between the Nrf2 expression score and WHO grade for astrocytic tumors (*p* = 1.8 × 10^−4^). Similarly, the average expression score of Nrf2 in anaplastic oligodendrogliomas was significantly higher than that in oligodendrogliomas (*p* = 0.026, Figure 4). In addition, the average expression score of Nrf2 in WHO grade III anaplastic ependymoma was higher than that of WHO grade II ependymoma. Our statistical analysis determined that ependymomas of more advanced WHO grades also had higher intensities of Nrf2 expression and greater percentages of Nrf2-positive tumor cells (*p* = 0.021). Therefore, among the included gliomas, Nrf2 expression scores in WHO grade III and IV tumors were significantly higher than those in WHO grade I and II tumors (*p* = 1.3 × 10^−5^, Figure 5A).

### 2.5. Nrf2 Expression Discriminates Atypical from Benign Meningiomas

**Figure 6:**
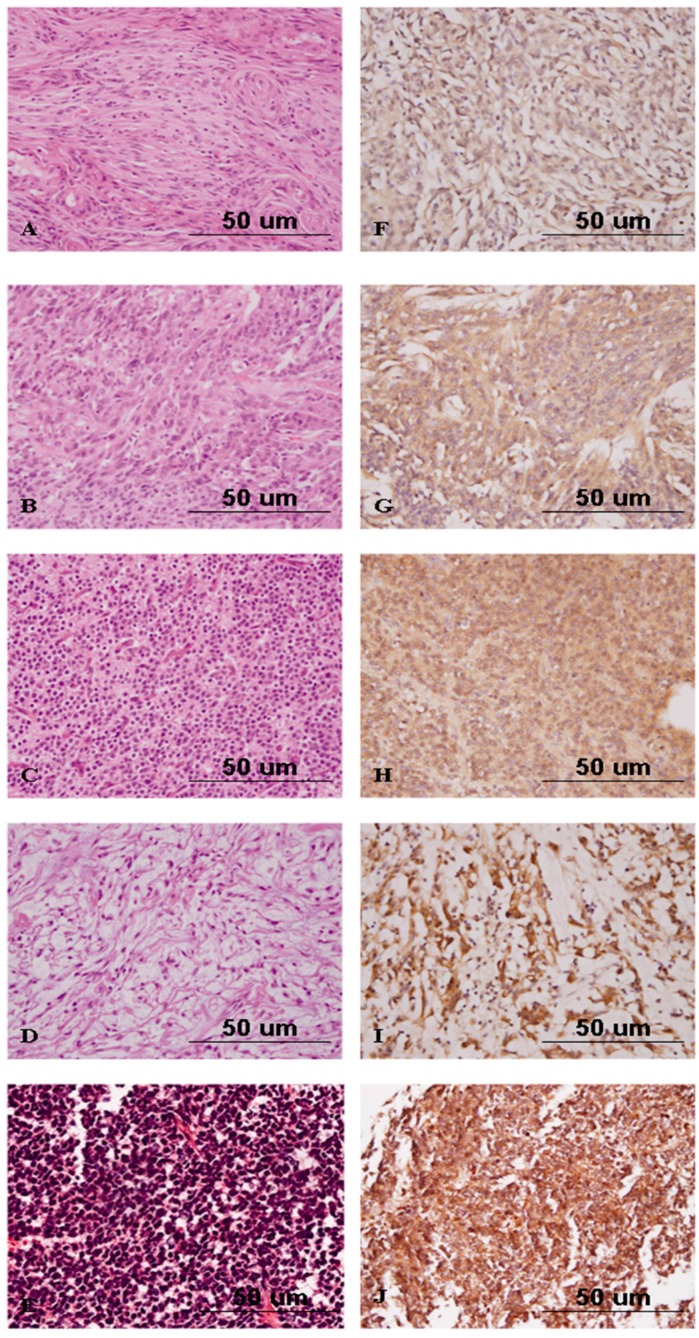
Hematoxylin and eosin staining of meningothelial meningioma (**A**); atypical meningioma (**B**); central neurocytoma (**C**); chordoma (**D**); and medulloblastoma (**E**); and immunohistochemical analysis of Nrf2 in meningothelial meningioma (**F**); atypical meningioma (**G**); central neurocytoma (**H**); chordoma (**I**); and medulloblastoma (**J**) (original magnification ×400).

Among the subtypes of WHO grade I meningiomas, the average expression scores of Nrf2 were 55.12 in tumors of the meningothelial subtype, 46.43 in those of the fibrous subtype, 53.00 in those of the psammomatous subtype, and 18.33 in those of the angiomatous subtype. Additionally, the Nrf2 expression score in atypical meningiomas was 133.33 (Table 3). The average Nrf2 expression score in each subtype of meningioma was higher than that in non-neoplastic brain tissues (Figure 5B). In addition, WHO grade II meningiomas had significantly stronger intensities of staining and greater percentages of Nrf2-positive tumor cells than did WHO grade I meningiomas (*p* = 1.8 × 10^−5^, Figure 6). Similarly, the average expression scores of Nrf2 were 99.29 in central neurocytomas, 47.50 in chordomas, and 170.00 in medulloblastomas. Expression of Nrf2 tended to be higher in WHO grade IV tumors than in WHO grade II tumors, even in PBTs other than gliomas and meningiomas (Table 4).

### 2.6. The Relationship between Nrf2 Imunostain Scores and Overall Survival Rates in Gliomas and Meningiomas

**Figure 7:**
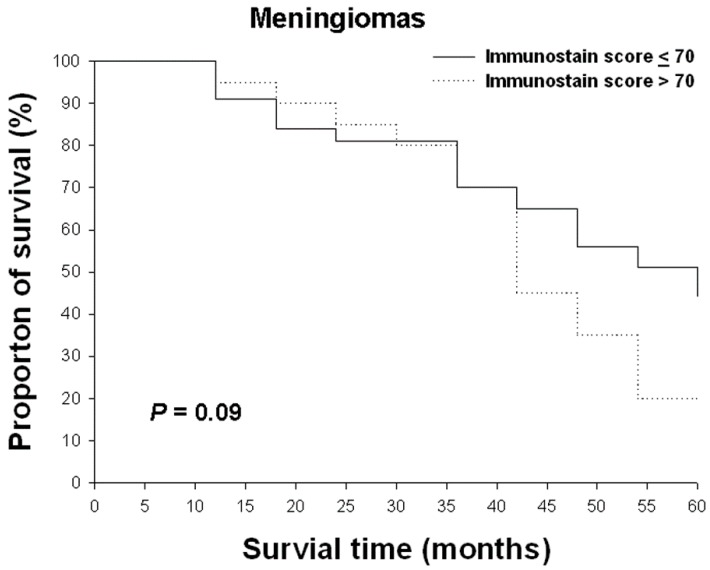
The relationships between the overall survival rate and Nrf2 immunostain scores in meningiomas. Survival rates were analyzed using the Kaplan–Meier survival test.

We divided the 63 with meningioma into two groups on the basis of Nrf2 expression, with a cut-off score of 70 for Nrf2 expression. These patients had been followed for five years. Higher Nrf2 expression tended to be associated with shorter survival for these patients, but the association did not reach statistical significance (Figure 7).

## 3. Discussion

Nrf2 has recently been found to be crucial for maintaining self-renewal in glioma stem cells [24,25]. In addition, Pan *et al.* [11] demonstrated that Nrf2 induces invasion and migration of human glioma cells. Nrf2 associates with the Bcl-xL and hypoxia-inducible factor-1α (HIF-1α) proteins, which promote cell survival and chemotherapeutic resistance in glioblastomas [12,26,27,28,29,30]. In addition, Boustani *et al.* demonstrated Nrf2 overexpression significantly correlated with age, tumor grade and overall survival rate in glioma patients [31]. However, the effects of Nrf2 expression on clinicopathologic parameters in other human PBTs are still unknown. Our research demonstrates that higher expression of Nrf2 is significantly correlated with more advanced WHO grade in gliomas and meningiomas. This result implies that Nrf2 might regulate aggressive behavior in human PBTs.

Currently, WHO histological grade is one of the most important prognostic factors in glioma and meningioma [4]. Unfortunately, to determine WHO grade in these tumors is sometimes not easy because of the limited specimen and complex cellular structure. An additional challenge when making a precise diagnosis in glioma is that it requires an accurate assessment of tumor invasion and proliferative activity [32]. Because of the differences in policies regarding treatment of low-grade (WHO grade I and II) and high-grade (WHO grade III and IV) gliomas, a precise determination of WHO grade is critical to provide proper treatment, prolong OS, and slow disease progression. In the current study, high-grade gliomas had significantly higher expression of Nrf2 than did low-grade gliomas. Our results indicate that characterization of Nrf2 expression could improve the accuracy of pathologists’ determination of WHO grade in glioma.

In our *in vitro* studies, we applied normal brain cell lysates as non-neoplastic brain tissue in the Western blot analysis. Although the heterogeneous components were included in these commercial lysates, purification of normal glial cell lysates from non-neoplastic brain tissue was difficult. In order to realize the target molecular protein expression of normal brain tissue, several studies used normal brain cell lysates instead of glial cell lines [33,34]. Otherwise, although gliosarcoma belongs to WHO grade IV gliomas, Nrf2 expression in gliosarcoma presented weaker intensity and less percentage than glioblastoma multiformes. The hypothesis was the biphasic histological patterns consisting of glial and sarcomatous components. Fibroblastic, smooth muscle, adipose tissue, cartilage and bone tissue might be included in the sarcomatous component of gliosarcoma [35]. Therefore, significantly lower Nrf2 expression was identified in the sarcomatous area than in glial region of gliosarcomas.

The notable histological evidence of low grade glioma depends on glial cell proliferation and mild nuclear enlargement. Additionally, gliosis is a non-specific astrocytosis on the repair period from brain injury. In the routine practice of surgical neuropathology, distinguishing between reactive gliosis and low-grade glioma is difficult. This is particularly true when a diagnosis is based only on histological images of the biopsy specimen [36]. In addition, the primary clinical factor associated with tumor recurrence in glioma is the degree of tumor clearance. The standard therapeutic method for low-risk, low-grade gliomas would be to excise the tumor with clear surgical margins. However, PBTs generally lack a landmark to label the edge of the tumor. In our study, the expression of Nrf2 in low-grade gliomas was significantly higher than that in non-neoplastic brain tissues. Because most of the included non-neoplastic brain tissues in this study revealed reactive astrocytosis, our results imply that immunohistochemical analysis of Nrf2 overexpression could not only help to discriminate glioma from gliosis in brain lesions, but also to discern the location of safe tumor margins in cases of PBT. Therefore, high expression of Nrf2 immunohistochemical stain might indicate the area of glioma involvement.

## 4. Experimental Section

### 4.1. Human Glioma Cell Lines and Lysates Preparation

U87MG, LN229, GBM8401 and U118MG human glioma cells lines were maintained in Duobecco’s modified Eagle’s medium (DMEM) containing 10% fetal boving serum (FBS), penicillin, and streptomycin. Glioma protein lysates were prepared from 2 × 10^7^ U87MG, LN229, GBM8401 and U118MG glioma cells. Normal brain tissue protein lysates were purchased from Origene Technologies.

### 4.2. Western Blots Analysis

All of included protein lysates were ready to use in Western blots analysis. Primary antibodies were a polyclonal rabbit anti-human Nrf2 antibody (Santa Cruz Biotechnology, Santa Cruz, CA, USA) and a monoclonal mouse anti-β-actin antibody (Sigma-Aldrich, St. Louis, MO, USA). The protocol of Western blot analysis was performed as previous study [33].

### 4.3. RNA Isolation and Real-Time Reverse Transcription-PCR

The procedures of total mRNA extraction, reverse transcription and quantitative RT-PCR were following previously protocol [33]. The normal brain cDNA was purchased from Origene Technologies (Rockville, MD, USA). In addition, the relative quantitative gene expression against an internal control, GADPH was performed using the 2^−ΔΔ*C*t^ method. The primer pairs used were: Nrf2 forward, 5′-ACACGGTCCACAGCTCATC-3′ and reverse, 5′-TGTCAATCAAATCCATGTCCTG-3′ [23], GADPH forward, 5′-CTTCATTGACCTCAACTAC-3′ and reverse 5′-GCCATCCACAGTCTTCTG-3′.

### 4.4. Tissue Microarray Construction

We constructed 2 tissue microarrays that included samples from 10 biopsies of non-neoplastic brain tissue, 92 cases of glioma of various WHO grades, 69 of meningiomas, 8 of central neurocytomas, 4 of chordomas, and 5 of medulloblastomas. The paraffin-embedded tumor cassettes of all of the cases included in the microarray were collected from 1991 to 2005. Each core on the microarray was 2 mm in diameter and was taken from a selected area of each paraffin-embedded tumor tissue. All non-neoplastic brain tissue blocks were collected from the patients with intracranical hemorrhage. The pathological diagnosis of all sampled cases was reviewed by at least 2 experienced pathologists. The histological differentiation of the PBTs was determined in accordance with the criteria of the WHO classification system for tumors of the central nervous system [4]. The tissue microarray slides displayed uniform staining that was equivalent to that of the original paraffin-embedded specimens. None of the included patients had ever received radiotherapy and/or chemotherapy before surgery.

### 4.5. Immunohistochemistry and Statistical Analysis

The tissue microarray sections were dewaxed in xylene, rehydrated in alcohol, and immersed in 3% hydrogen peroxide for 5 min to suppress endogenous peroxidase activity. Antigen retrieval was performed by heating each section to 100 °C for 30 min in 0.01 mol/L sodium citrate buffer (pH 6.0). After 3 rinses (each for 5 min in phosphate buffered saline (PBS)), the sections were incubated for 1 h at room temperature with a polyclonal rabbit anti-human Nrf2 antibody (1:50, Santa Cruz Biotechnology, Santa Cruz, CA, USA) diluted in PBS. After 3 washes (each for 5 min in PBS), the sections were incubated with biotin-labeled secondary immunoglobulin (1:100, DAKO, Glostrup, Denmark) for 1 h at room temperature. After 3 additional washes, peroxidase activity was developed with 3-amino-9-ethylcarbazole substrate chromogen (DAKO, Glostrup, Denmark) at room temperature.

To score Nrf2 expression in each sample, we assessed both the number of tumor cells that expressed Nrf2 and the intensity of staining. Any samples with more than 5% of tumor cells displaying cytoplasmic and nuclear staining were considered positive. The intensity of Nrf2 staining in tumor cells was scored on a scale of 0 to 3, with 0 being absence of staining; 1, weak staining; 2, moderate staining; and 3, strong staining. Weak, moderate, and strong cytoplasmic and nuclear staining was classified by using microscopy at a magnification of 40×, 20×, 10×, and 4×, respectively [36]. The percentage of positive cells (from 5 to 100) was multiplied by the corresponding average immunostaining intensity (from 0 to 3) to obtain an Nrf2 expression score, which ranged from 0 to 300. The expression of Nrf2 in samples from endometrial adenocarcinoma was used as a positive control [37]. Statistical analysis was performed by using the Pearson Product Moment method. A correlation of WHO grade with expression score was established if the *p* value was less than 0.05.

### 4.6. Overall Survival Rate Calculation

Patient survival was calculated from the date of surgery to the date of death. The OS for patients diagnosed with 63 cases of meningioma was recorded after a 5-year follow-up. The relationship between OS and the Nrf2 expression score was analyzed by using the Kaplan–Meier survival test.

## 5. Conclusions

In conclusion, Nrf2 is a potential biomarker that can be used not only to detect the neoplastic component of small and poorly oriented specimens, but also to predict WHO grade in PBTs. The combination of immunohistochemical analysis of Nrf2 expression and a hematoxylin and eosin stain will assist pathologists in making a conclusive and accurate diagnosis. Patients whose glioma or meningioma had higher Nrf2 expression tended to have lower OS, although the association did not reach statistical significance. This lack of significance may be due to the small number of glioma and meningioma cases with long-term follow up, which could result from the short OS of many patients. Although the details of pathogenesis in PBT remain unclear, pharmacological targeting of Nrf2 could delay disease progression and tumor infiltration.

## Figures and Tables

**Table 1:** The classification of brain tumors and World Health Organization (WHO) grading of 178 cases.

Classification of Tumors	Numbers
**Meningioma**	
Meningothelial meningioma (WHO grade I)	42
Fibrous (fibroblastic) meningioma (WHO grade I)	7
Psammomatous meningioma (WHO grade I)	5
Angiomatous meningioma (WHO grade I)	3
Atypical meningioma(WHO grade II)	12
**Astrocyitc tumors**	
Pilocytic astrocytoma (WHO grade I)	7
Diffuse astrocytoma (WHO grade II)	41
Anaplastic astrocytoma (WHO grade III)	11
Glioblastoma multiforme (WHO grade IV)	9
Gliosarcoma (WHO grade IV)	4
**Oligodendroglial tumors**	
Oligodendroglioma (WHO grade II)	6
Anaplastic oligodendroglioma (WHO grade III)	9
**Ependymal tumors**	
Ependymoma (WHO grade II)	3
Anaplastic ependymoma (WHO grade III)	2
**Other brain tumors**	
Central neurocytoma (WHO grade II)	8
Chordoma (WHO grade II)	4
Medulloblastoma (WHO grade IV)	5

**Table 2:** The intensity of Nrf2 immunostaining and clinicopatholigcal parameters of gliomas.

Classification of Tissue	Average Intensity	Average % Tumor	Average Score	Correlation *
**Normal brain tissue**	0.67	12.78	12.78	-
**Classification of gliomas**				
Pilocytic astrocytoma	0.43	19.29	19.29	Positive correlation (*p =* 1.8 × 10^−4^)
Diffuse astrocytoma	1.29	51.95	75.98	
Anaplastic astrocytoma	2.1	67.27	114	
Glioblastoma multiforme	1.89	83.33	160.56	
Gliosarcoma	1.25	32.5	42.5	
Oligodendroglioma	1	55	71.67	Positive correlation (*p =* 0.026)
Anaplastic oligodendroglioma	2.11	77.78	171.11	
Ependymoma	1	70	70	Positive correlation (*p =* 0.021)
Anaplastic ependymoma	2.5	85	212.5	
**WHO grades of gliomas**				
WHO grade I	0.43	19.29	19.29	Positive correlation (*p =* 1.3 × 10^−5^)
WHO grade II	1.27	55.21	77.81	
WHO grade III	2.21	75.79	173.42	
WHO grade IV	1.69	67.69	124.23	

* The correlation was analyzed by Pearson Product Method Correlation test.

**Table 3:** The intensity of Nrf2 immunostain scores and clinicopatholigcal parameters of meningiomas.

Classification of Tissue	Average Intensity	Average % Tumor	Average Score	Correlation *
Classification of tumors				
Meningothelial meningioma	1.05	41.67	55.12	-
Fibrous meningioma	0.86	46.43	46.43	-
Psammomatous meningioma	1	53	53	-
Angiomatous meningioma	0.67	18.33	18.33	-
Atypical meningioma	1.75	70.83	133.33	-
**WHO grades of meningiomas**				
WHO grade I	1	42.02	51.93	Positive correlation (*p =* 1.8 × 10^−5^)
WHO grade II	1.5	67.91667	105.8333	

* The correlation was analyzed by Pearson Product Method Correlation test.

**Table 4:** Nrf2 immunostain scores in other neuroepithelial tumors.

Classification of Other Neuroepithelial Tumors	Average Intensity	Average % Tumor	Average Score	WHO Grades
Central neurocytoma	1.57	47.86	99.29	II
Chordoma	1.5	22.5	47.5	II
Medulloblastoma	2.2	78	170	IV
